# Practical Notes and Formulæ

**Published:** 1887-02

**Authors:** 


### PRACTICAL NOTES AND FORMULÆ.

**Whooping Cough.**—

*Editors Record:*

Have you anything late and good for whooping cough? I would be glad to hear from our medical brethren on the subject through the RECORD. J. T. S.

A medical friend now in our sanctum suggests the following as a very superior remedy:

℞. Brom. pot.................................ʒ ss,

Brom. sodium.............................℥ j,

Brom. ammoni.............................ʒ ss.

Syrup chloral............................℥ jss,

Water....................................℥ ij.

M. Dose, one dessert spoonful in a little milk one to three times a day to a child one year old. If the white of an egg be added to the mixture it will improve it.

**For Vomiting.**—

℞. Acetic acid.............................gtts. xl,
Creasote...................................gtts. xx,
Sulph. morph...............................grs. ij,
Distil. water..............................℥ ij.

M. Sig.—One teaspoonful.

**Elixir of Iron Pyrophosphate, Quinine and Strychnine.**—Dr. C. R. Bechmann, of Fountain City, Wis., says, (*Medical and Surgical Reporter*) that an elixir prepared after the following formula will not precipitate by long standing:

℞. Sulphate of quinine........................grs. cxx,
Sulphate of strychnine.....................grs. ij,
Pyrophosphate of iron......................grs. ccxl,
Citrate of sodium or ammonium..............grs. lx,
Alcohol...................................fl℥ ij,
Water.....................................fl℥ j,
Glycerin..................................fl℥ iij,
Simple elixir, q. s. ad...................fl℥ xvi.

Put the quinine and strychnine in a flask, pour on the alcohol and 5 fluidounces of simple elixir, then place the flask in hot water, shaking occasionally until dissolved. Dissolve the iron in the water without heat, then add the citrate of sodium (or ammonium) and the glycerin. Pour this solution into the alkaloid solution flask, and shake well. When cold, add enough simple elixir to bring the quantity up to 16 fluidounces. An elixir made three months ago, and subjected to 41° F., has remained as clear as when first made.

**Eulylyptol.**—According to the *Journal of the American Medical Association*, eulylyptol is the name given by Dr. Schmeltz to a mixture containing 6 parts salicylic acid to 1 part each of carbolic acid and eucalyptol, and which he considers a better antiseptic than iodoform, corrosive sublimate, or carbolic acid. A small quantity added to urine will preserve it for months.

**Use of Lanolin in Midwifery Practice.**—We notice the following in the *Cincinnati Medical News:* "Dr. C. Clark Burman calls our attention through the *Lancet* to lanolin as a particularly efficient means of reducing the ridgity of the perineum in primiparæ. Owing to its penetrative and softening properties, he finds its much preferable to the plan adopted of friction with pure lard. Only recently this ridgity was strongly marked in an elderly primipara, where the forceps were required on account of debility on the part of the patient. He was surprised with what facility he succeeded in reducing it, so as to enable him to apply the forceps much sooner than he should otherwise have felt justified in doing, on account of the danger of severe laceration."—*Druggist.*

**Biniodide of Mercury in Diphtheria and Scarlatina.**—Dr. Turner, in the *Medical Age*, says: In four years I have used it in more than eighty cases of diphtheria and never lost a case.

℞. Merc. biniodide.............................gr. j,
Sacch. pepsin, P., D. & Co......................ʒ ij.

M. Ft. ch. no. xx. Sig.—one every two hours.

**Conjunctivitis.**—Dr. Thomas, in *Medical Progress*, says: In simple conjunctivitis (not specific) I have found no collyrium better than the following:

℞. Liquid ergotæ (normal)..........................ʒ ij,

Hydrastini.................................grs. v,

Aquæ destill..................................℥ j.

M.

Often the hydrastin is omitted, and results are good. In some forms of ophthalmia, not specific, accompanied with excessive secretion, ether lachrymal or muco-purulent, it is almost a specific; of course, as is usually the case, when there is much pain some anodyne, as morphia, is added—more recently ʒ j of a 4 per cent. sol. of cocaine.

**Bi-sulphide** of carbon kills microbes, arrests fermentation, and is an all-powerful antiseptic. It has been successfully administered in France, in cases of typhus, to disinfect the breath and excretions of the patients. It also arrests diarrhœa. It is employed in aqueous solution.—*Med. World.*

**For Headache of Dysmenorrhœa.**—

| | | | |
|---|---|---|---|
| ℞. | Chloral hydr | dr. ij, | |
| | Morphine sulph | gr. ss, | |
| | Syrup simp... } Aqua font..... } aa | oz. ss. | M. |

Sig. A teaspoonful every hour until quiet.

**Tonic for Children.**—

| | | | |
|---|---|---|---|
| ℞. | Ferri phosphat | gr. iij, | |
| | Pulv. Cinnam Co | gr. v. | M. |

Sig. At a draught.—*Ex.*

**Tonic in Phthisis.**—

| | | | |
|---|---|---|---|
| ℞. | Ferri citrat | gr. v, | |
| | Quin. sulph | gr. j, | |
| | Acidi citric | gr. x, | |
| | Aquæ ad | ℥ i. | M. |

Sig. To be taken with 10 grains of bicarbonate of soda.—*Ex.*

**Chronic Diarrhœa.**—

| | | |
|---|---|---|
| ℞. | Pulv. calumb | ℥ i, |
| | Pulv. zinzib | ʒ ii, |
| | Aquæ bullientes | O j. |

In funde per horas duas, et cola.

S. A wineglassful, cold, every two hours.—*Dr. Ellis.*

**Chronic Skin Eruptions.**—

Dr. James Eakins, of Port Austin, reports to the Medical Age good success with the following :

| | | | |
|---|---|---|---|
| ℞. | Liq. pot. arsenit | gtt. ij, | |
| | Ferri cit | gr. ij, | |
| | Pot. iodid | gr. v, | |
| | Syr. glycer, aquæ, aa | ʒ ss. | M. |

Sig. At one dose, three times a day.

**Infantile Colic.**—Professor Bartholow (New England Medical Monthly) recommends—

| | | | |
|---|---|---|---|
| ℞. | Potassii bromidi | ʒ i, | |
| | Ol. anisi | *m* i, | |
| | Mucil. acacia | ℥ i, | |
| | Aqua munth. pip | ℥ i. | M. |

Sig. One teaspoonful every half hour.

**Chronic Bronchitis.**—New England Medical Monthly :

| | | | |
|---|---|---|---|
| ℞. | Tinct. capsici | ʒ ss, | |
| | Tinct. nucis vomicæ | ʒ ij, | |
| | Tinct. sanguinariæ | ʒ i, | |
| | Vini picis | ℥ iv. | M. |

Sig. Dessertspoonful four times a day.

